# Early Effects of a Pain-Informed Movement Program in Patients with Post-COVID-19 Condition Experiencing Persistent Pain: Protocol for a Randomized Controlled Trial

**DOI:** 10.3390/jcm13020597

**Published:** 2024-01-20

**Authors:** Andrés Calvache-Mateo, Laura López-López, Alejandro Heredia-Ciuró, Javier Martín-Núñez, Geraldine Valenza-Peña, Irene Cabrera-Martos, Marie Carmen Valenza

**Affiliations:** Department of Physiotherapy, Faculty of Health Sciences, University of Granada, Av. De la Ilustración, 60, 18016 Granada, Spain; andrescalvache@ugr.es (A.C.-M.); lauralopez@ugr.es (L.L.-L.); ahc@ugr.es (A.H.-C.); javimn@ugr.es (J.M.-N.); geraldinevalenza@ugr.es (G.V.-P.); cvalenza@ugr.es (M.C.V.)

**Keywords:** post COVID-19 condition, Pain Informed Movement program, protocol, persistent pain, intervention, randomized control trial

## Abstract

(1) Background: The COVID-19 pandemic has generated 771 million confirmed cases. Of these patients, 60% have developed persistent symptoms including pain. This pain is a complex symptom that needs comprehensive therapeutic strategies to address it holistically. The main objective of this study will be to evaluate the early impact of the Pain Informed Movement (PIM) program in patients with post-COVID-19 conditions experiencing new-onset persistent pain. (2) Methods: A randomized, single-blind clinical trial will be performed. Patients will be randomly assigned (1:1) to the experimental or control group. The experimental group will undergo a PIM program consisting of low-intensity functional exercises, pain neuroscience education, and relaxation techniques. The control group will receive no intervention. (3) Results: The results will be published as a peer-reviewed article. (4) Conclusions: This study will provide a basis for future research to support the implementation of comprehensive therapeutic approaches in the care of patients with post-COVID-19 persistent pain.

## 1. Introduction

The COVID-19 pandemic has represented a major medical challenge with potential long-term repercussions [1,2]. To date, more than 771 million confirmed cases have been reported [3], and approximately 60% of these patients develop persistent symptoms [4,5].

Patients are considered to have post-COVID-19 conditions when symptoms last more than 3 months [6]. Among these symptoms, post-COVID-19 persistent pain emerges as a frequent manifestation that affects the quality of life and functionality of those suffering from this condition.

Fernandez-de-las-Peñas et al. carried out an exhaustive analysis that revealed a 10% incidence of post-COVID-19 persistent pain [7]. However, previous research focused on the study of this symptom shows a frequency of 45–70% [8,9,10,11,12,13,14,15,16]. These data indicate that the magnitude of persistent pain may be underestimated in overall studies of post-COVID-19 condition patient cohorts [10,16].

The evidence available so far indicates that the experience of persistent pain in post-COVID-19 condition patients follows a nociplastic pain model [17,18]. Nociplastic pain is defined by an exacerbated sensitivity to pain and is characterized by a chronic central sensitization process that is enhanced over time [19,20] by symptoms related to the central nervous system such as psychological distress, mood disturbances, and sleep disorders [21,22,23,24].

Post-COVID-19 persistent pain presents a unique complexity, with multiple contributing factors beyond the initial respiratory implications. The patient-centered approach [25,26] based on an understanding of pain as a multifaceted phenomenon, reflects the need for holistic therapeutic interventions to address post-COVID-19 persistent pain.

To address this complexity, the need arises for comprehensive therapeutic strategies that not only address the painful symptomatology but also address potential implications at the neuromuscular and psychological levels. In this context, the Pain Informed Movement (PIM) [27] program is presented as a novel approach that combines low-intensity functional exercises, relaxation techniques, and pain neuroscience education.

The main objective of this study will be to evaluate the early impact of the PIM program in patients with post-COVID-19 conditions experiencing new-onset persistent pain. Through the implementation of this program, we seek to determine whether such intervention can offer significant improvements in reducing pain intensity and interference, catastrophizing, kinesiophobia, and functionality in patients affected with post-COVID-19 conditions.

## 2. Materials and Methods

### 2.1. Design

The study will be a randomized, single-blind clinical trial to analyze the effect of the PIM program on patients with post-COVID-19 conditions experiencing persistent pain. This group will be compared with a control group. The research assistant collecting the data will be blinded to the hypothesis of the study and the patient’s allocation. Because of the nature of the intervention, it will not be feasible to blind the participants. The protocol of this study has been registered in Clinicaltrials.gov: NCT05475743.

### 2.2. Ethical Considerations

The study protocol was approved by the Local Research Ethics Committee with registration number 2392/CEIH/2021. It will be conducted following the 1975 Declaration of Helsinki, as revised in 2013 [28], and the Consolidated Standards of Reporting Trials (CONSORT) [29]. An informed consent form will be signed by the participants after being informed about the study conditions.

### 2.3. Setting

This study will be conducted at the Faculty of Health Sciences of the University of Granada (Spain). Participants will be recruited from the Long-COVID Regional Association (Long-COVID Andalucía).

### 2.4. Participants

This study will include patients over 18 years of age with a diagnosis of post-COVID-19 with new-onset moderate or severe pain. According to the World Health Organization (WHO) definition, patients present post-COVID-19 condition when they have persistent symptoms up to 3 months after the onset of the disease, lasting at least 2 months with no alternative diagnosis that explains these symptoms [6]. In addition, moderate or severe pain not limited to a specific anatomical district or origin will be assessed with a score greater than or equal to 3.5 using a Visual Analog Scale (VAS) [30]. Patients will be excluded if they present any of the following conditions: pulmonary, cardiac, neurological, vascular, or orthopedic pathologies that may limit the performance of the assessment and the intervention, cognitive impairment that prevents them from understanding and answering the questionnaires, or if they suffer reinfection by SARS-CoV-2. In addition, those patients with a history of severe or critical illness due to COVID-19 [6] and with pre-existing chronic pain according to the current International Association for the Study of Pain definition [31,32] will be excluded. Finally, patients who participated in other studies will also be excluded.

### 2.5. Randomization

Participants in this study will be randomly assigned to two groups: the experimental group and the control group. The eligibility of participants will be assessed by an impartial professional who will not be involved in the randomization process. The randomization will be performed by an independent researcher who will not be involved in the evaluation or treatment of participants. The patients will be assigned to their respective groups sequentially. A computer program (https://www.randomizer.org) (accessed on 1 August 2023) will be used to generate a 1:1 ratio randomization sequence. The allocation sequence will be generated and maintained off-site at the study site. The information on the allocation of patients to the intervention or control group will be transmitted by mail to the recruiter. The evaluation will be carried out by a physical therapist blinded to treatment assignments.

### 2.6. Intervention

The experimental group will be included in an 8-week PIM program. This program will include pain neuroscience education, functional exercise, consisting of low-intensity strengthening exercises, and relaxation techniques. The Template for Intervention Description and Replication (TIDieR) checklist [33] will be used. The control group will receive a leaflet. Patients in both groups will be allowed to take pharmacological treatment (paracetamol or non-steroidal anti-inflammatory drugs) if needed and under the prescription of a physician. Patients’ evaluations will be conducted at baseline (T1) and immediately post-intervention (T2).

#### 2.6.1. Experimental Group

The patients included in the experimental group will attend an 8-week PIM program in addition to standard medical care. The program is based on a previous study [27] and will be led by a physical therapist with experience in treating patients with chronic pain. This program will consist of 2 face-to-face sessions per week, and they will be asked to complete two sessions at home weekly. The first face-to-face session will be a group session with 3–6 participants, and the second face-to-face session will be individual. Both sessions will last approximately 1 h. In the group sessions, pain neuroscience education will be conducted. In the individual sessions, an individualized functional exercise program will be performed by each patient, as well as relaxation techniques. Patients will be asked to perform the functional exercises and relaxation techniques at home. Participants will be asked to record their compliance with the program and their progress.

Education in pain neuroscience is considered an important pillar in the comprehensive approach to persistent pain. Multiple studies support its use in patients with this complex symptom. This technique will allow the patient to better understand the complexity of the nervous system and the pain process. In this way, education in pain neuroscience will allow the therapist to provide the patient with the cognitive tools necessary to understand and adequately manage pain.

Pain neuroscience education will consist of group sessions in which simple explanations will be given on the difference between tissue damage and pain, especially in the case of chronic pain, central nervous system level nociception processing, pain modulatory signals, neuroplasticity and adaptability of other systems, and the relationship between stress, thoughts, emotion, and pain. The fundamental objective of this technique is to explain to the patient how the human being processes and perceives pain. The use of this technique will help the patients to understand their own experiences. Improving the patients’ knowledge of pain allows them to discard all the stigmas associated with persistent pain and will give them tools that will allow them to be active in their treatment.

The first point when applying this technique in patients with persistent pain is to disprove all the inherited erroneous beliefs that the patient may have. The main erroneous belief that must be disproved is the association between pain and tissue damage. The aim is to make the patient understand that pain is a multifaceted phenomenon, influenced by physical, cognitive, and emotional factors. Once the patient understands this, he/she will be able to break the cycle of anxiety and stress associated with persistent pain.

The second fundamental point of this educational technique is the explanation of the concept of brain plasticity. The assimilation of this concept by the patient will allow him/her to understand that the nervous system is not something static but can modify itself over time.

The third and last point is to provide patients with the necessary tools to address the emotional problems that are linked to persistent pain. The connection between catastrophizing, kinesiophobia, and persistent pain is explored, thus allowing an improvement in mental health associated with better management of persistent pain. This new perspective for patients gives them hope and confidence in adopting therapeutic strategies that will enable them to manage pain in the long term. In this way, persistent pain is no longer an experience full of unknowns and doubts, but a challenge that can be addressed.

The functional exercise program will consist of 3 different parts. The first part will be a warm-up focused on joint mobilization. The second part will focus on functional exercises consisting of low-intensity strengthening exercises of the main muscle groups. This section will have four main objectives: core stability, postural positioning, limb muscle strength, and functional activities. The proposed exercises have been previously used in this population demonstrating their reliability and safety [34,35]. Some of them are the bodyweight squat, hip thrust, rowing, front plank, lateral squat, and chest press. However, the exercises, the number of sets and repetitions, and rest periods will be individually adapted to each patient depending on their needs, severity of pain, and physical ability with different levels of progression. The exercises will be adapted so that they can be performed at home without the need for specific materials. Only when the patient can perform the exercises with the correct technique and without effort, the exercises will be progressed to more demanding exercises or a higher training load.

Finally, relaxation techniques will be performed. Relaxation strategies will include practicing mindfulness and breathing control, cultivating body awareness and muscle tension management, as well as awareness of thoughts and emotions related to pain.

The mindfulness practice will focus on trying to make the patient aware of the present moment. To do this, the patient will be asked to focus their attention on the bodily sensations they have at that moment and on their breathing rhythm. The goal is to allow the patient to disconnect from the persistent pain, focusing on healthy sensory experiences. Breathing control is a fundamental technique to improve the stress response. Participants should practice deep and conscious breathing, which will allow a more efficient oxygen intake and carbon dioxide output, thus contributing to reducing the activation of the sympathetic nervous system and therefore the perception of pain.

The body awareness and muscle tension management techniques will be performed through exercises that allow patients to develop a greater awareness of areas of tension. Increased body awareness will allow patients to become proactively involved in pain management.

Finally, cognitive change techniques will be used to improve pain-related emotions. Patients will be asked to identify negative thought patterns and redirect them towards more positive and constructive thoughts. The aim of performing these techniques is to contribute to a better management of the psychological impact of persistent pain.

The set of relaxation techniques is one of the essential components of this intervention program. It is intended that these techniques not only help to alleviate the painful symptomatology but also to promote proactive changes, improving the patient’s self-regulation and quality of life, thus helping to generate a better overall experience.

During the face-to-face sessions, participants will be asked to perform the functional exercises and relaxation techniques at home. They will also be asked to record this so that the therapist can follow up properly. Participants will be free to ask any questions during the program.

#### 2.6.2. Control Group

In addition to standard medical care, patients assigned to this group will receive a leaflet with information about the main post-COVID-19 condition symptoms. This is a control group that will not be controlled or monitored by a therapist.

### 2.7. Outcomes

Patients will be initially contacted by telephone. Patients will be informed of the study and if they agree to participate a face-to-face appointment will be arranged. The initial evaluation will be performed once informed consent is obtained. The sociodemographic characteristics collected will include the anthropometric data, weeks since infection, percentage of smokers, comorbidities assessed with the Charlson comorbidities index [36,37], physical activity through the International Physical Activity Questionnaire Short Form (IPAQ-SF) [38] and working activities by self-report. In addition, patients will be asked to complete a diary with the pharmacological pain treatment they are taking.

#### 2.7.1. Primary Outcomes

Pain intensity and interference will be quantified using the Brief Pain Inventory (BPI). The section intended to measure pain intensity on the BPI comprises four items, while the section related to pain interference includes seven items. In the intensity section, responses range on a scale from 0 (no pain) to 10 (maximum pain), while in the interference section, responses range from 0 (no interference) to 10 (total interference). To calculate the severity and interference index, the values of the corresponding items are averaged, obtaining values ranging from 0 to 10, with a higher score indicating greater pain intensity and interference. In addition, body spatial pain distribution will be marked on a predetermined diagram provided in this inventory. The BPI has been validated as a reliable and valid tool to evaluate these aspects of pain [39,40]. The Spanish version of this scale has demonstrated high internal consistency, with an alpha coefficient of 0.93 [41].

To evaluate the phenomenon of catastrophizing, the Pain Catastrophizing Scale (PCS) will be applied [42]. This instrument consists of 13 statements structured on a 5-point Likert scale ranging from 0 (at no time) to 4 (at all times). The PCS breaks down catastrophizing into three components: feelings of helplessness, magnification of difficulties, and rumination about pain [43,44]. Each of the items describes different thoughts and emotions that people may experience when in pain. The final score is obtained by summing the scores of all the items, resulting in a score ranging from 0 to 52, with higher scores indicating a higher level of catastrophizing. It is important to note that the Spanish version of this scale, used in this study, has demonstrated adequate internal consistency, with an alpha coefficient of 0.79 [45].

The Spanish translation of the Tampa Kinesiophobia Scale (TSK) will be used to quantify apprehension related to physical activity and the fear of injury (or re-injury). This scale is composed of 11 statements, which are assigned a score on a 4-point scale (1–4). Total scores vary between 11 and 44 points, with higher values being indicative of greater fear of movement and the possibility of (re)injury. In the Spanish version of this scale, a moderate internal consistency has been found, with a coefficient alpha of 0.79 [46].

Functionality will be assessed using the WHODAS 2.0 tool (World Health Organization Disability Assessment Schedule) [47], using the Spanish version [48,49]. It consists of 36 items assessing six main areas of functional ability. This questionnaire uses a 5-point Likert scale for respondents to rate their level of difficulty in six main subscales: Cognition, Mobility, Self-Care, Interpersonal Relationships, Home Life, and Community Involvement. Response options go from 1 (no difficulty) to 5 (extreme difficulty or can not do). The scores obtained in each area are added to obtain a total score that shows the degree of functional limitation.

#### 2.7.2. Covariables

Anthropometric data such as age, gender, and body mass index, and clinical data such as weeks since infection, and comorbidities assessed with the Charlson comorbidities index [36,37] will be recorded.

### 2.8. Sample Size Calculation

An a priori sample size was based on the minimum clinically important difference (MCID) of the BPI interference estimated to be 1.00 [50]. Using G*Power 3.1.9.2, we obtained a sample size of 49 participants, equally distributed between the treatment and control groups, based on a confidence level of 95% (α = 0.05) and a power of 80% (β = 0.2). Anticipating a 20% dropout rate, the total sample size was increased to ensure adequate statistical power and the resulting sample size was 30 participants per group.

### 2.9. Statistical Analysis

Data will be analyzed using the Statistical Package of Social Science (SPSS) program for Windows (version 26 IBM, Armonk, NY, USA). Categorical data will be expressed as frequency (percentage). Continuous variables will be presented as mean and standard deviation (SD). The normality of the data will be first tested with the Shapiro-Wilk test. Characteristics of both groups will be compared using the Chi-square test for categorical variables. For continuous variables, the Student *t*-test will be used for those with a normal distribution and the Mann-Whitney U test for nonparametric variables. All effect sizes will be interpreted according to Cohen’s conventions: 0.3 = small, 0.5 = moderate, and 0.8 = large. A per-protocol analysis will be performed including participants who completed the treatment plan. Statistical significance will be set at *p* < 0.05.

## 3. Results

The results will be published as a peer-reviewed article. The authors intend to generate 3 tables summarizing the main results of this article. The first will be the table of descriptive characteristics of the sample. The second table will present the data of the comparison between groups at baseline for the primary outcomes. The third table will present the post-intervention outcome data, showing the intragroup and between-group changes, and expressing the effect size using Cohen’s d. In addition, a figure with the flow diagram will be added.

## 4. Discussion

The objective of this study will be to evaluate the early effects of the PIM program in post-COVID-19 condition patients experiencing persistent pain on pain intensity and interference, catastrophizing, kinesiophobia, and functionality. The results of this study are expected to demonstrate the effectiveness of the PIM program in reducing pain intensity levels and pain interference with daily activities compared to the control group. In addition, the results are expected to demonstrate the efficacy of the PIM program in reducing levels of catastrophizing, and kinesiophobia, as well as increasing levels of functionality. By conducting this study, we aim to provide clinicians with an intervention program with a comprehensive ability to address both the physical and psychological aspects of post-COVID-19 persistent pain.

The sample of subjects included in this study is expected to be similar to that of previous studies performed in post-COVID-19 patients, with similar anthropometric characteristics [51,52]. The higher prevalence of post-COVID-19 conditions in the female gender has been previously proven. These disparities in prevalence arise due to different symptomatic, inflammatory, and immunologic responses between men and women [53,54]. In addition, previous studies conducted on patients with post-COVID-19 conditions show that most patients with this condition are middle-aged, with median ages ranging from 45–55 years [7]. Thus, the sample included in this study is expected to include a higher proportion of women and have an average age of about 50 years.

Although the published scientific evidence demonstrates the high prevalence of persistent pain in patients with post-COVID-19 conditions [55] and highlights the importance of future research efforts to focus on this aspect [56], to the authors’ knowledge this will be the first randomized control trial that focuses on developing an intervention to specifically address this symptom. To date, only one study in patients with post-COVID-19 conditions has included pain as a variable. The article by Hausswirth et al. [57] shows a significant improvement in the intervention group, which in this case underwent a four-week neuro-meditation intervention, compared to the control group. However, in other studies conducting pulmonary rehabilitation [58,59] the pain subscales show no improvement. These results highlight the need for specific rehabilitation programs that address the complexity of this symptom.

Regarding kinesiophobia, Nambi et al. [60] demonstrated that low-intensity aerobic exercise is more effective in improving kinesiophobia than high-intensity aerobic exercise in community-dwelling elderly with post-COVID-19 sarcopenia. However, it is important to note that both groups of patients underwent a program of low-intensity functional exercises similar to the one that will be carried out in our article. A previous study showed that athletes report less intense pain than sedentary individuals in response to an experimental pain procedure, catastrophizing a significant predictor of pain. However, catastrophizing was not a mediator in pain levels between athletes and sedentary individuals [61]. Additionally, Giacomo et al. highlight the relevance of combining rehabilitation and specific training considering the biomechanics of musculoskeletal pain [62]. Therefore, although more studies are needed to confirm it, low-intensity functional exercise seems to be a good therapeutic strategy to improve this variable in patients with post-COVID-19 conditions. Moreover, catastrophizing values will be collected in both groups.

With respect to functionality outcomes, many interventions have been developed to date to try to improve this variable in patients with this condition. These interventions are respiratory rehabilitation programs focused mainly on respiratory, functional, and quality of life variables. Some of these interventions have been shown to be beneficial in improving patient functionality [63,64,65,66]. In contrast, other studies have not found these differences in favor of the intervention group [67,68,69].

In addition to respiratory rehabilitation interventions, symptom control interventions have been developed in patients with post-COVID-19 conditions. In this type of intervention, the results regarding functionality are not consistent. In the study by Philip et al. [57] no significant improvements were found in the intervention group with respect to the control group. In contrast, in the study by Kuut et al. [70] significant differences were found with respect to the control group, but with a small effect size (Cohen’s d = 0.34). Further studies are needed to determine whether these types of interventions can help improve the functionality of patients with this condition.

The PIM program has been previously used in patients with osteoarthritis showing to be effective in reducing pain levels and functionality [27]. The results of our study are expected to achieve minimal detectable change for the Brief Pain Inventory and WHODAS 2.0 tools with complex scoring. If the expected results are achieved the PIM intervention program will prove to be effective in the management of persistent pain in patients with post-COVID-19 conditions.

Despite the positive results, this study has some limitations. Although the intervention proposed in this article fits the complexity of persistent pain post-COVID-19, the fact that it is a comprehensive intervention will make it difficult to determine the exact mechanisms behind patients’ improvement. Our intervention will include low-intensity functional exercise, relaxation exercises, and education in pain neuroscience, so we should consider the possible contribution of each component of the intervention. It would be interesting to design future research to investigate the efficacy of the different components of the overall intervention. Furthermore, the lack of participant blinding due to the nature of the intervention could introduce bias.

The methodological strengths of the research stand out in the detailed planning of the intervention protocol, which is easily reproducible in clinical settings. The individualized adaptation of the PIM, considering the physical limitations and progress of each participant, adds a distinctive element. Random allocation and blindness of the research assistant to the study hypothesis and patient assignment strengthen the internal validity of the study design. In addition, the inclusion of patients with moderate or severe pain (VAS ≥ 3.5 cm) and the exclusion of those with limiting medical conditions improve the homogeneity of the sample and the applicability of the results. In addition, for a better analysis of the PIM program, a record will be made of the adverse effects as well as the number of sessions completed by the study participants to assess feasibility and adherence.

## 5. Conclusions

In conclusion, this study aims to demonstrate the efficacy of the PIM program in the management of persistent pain in patients with post-COVID-19 conditions. The expected results of this study aim to demonstrate that the PIM program not only relieves pain but also improves functionality and addresses the associated psychological aspects. Despite the limitations, the conduct of this study will provide a basis for future research to support the implementation of comprehensive therapeutic approaches in the care of patients with post-COVID-19 persistent pain.

## Data Availability

Not applicable.
